# A Rapidly Enlarging Asymptomatic Parapneumonic Effusion: A Case Report

**DOI:** 10.7759/cureus.52986

**Published:** 2024-01-26

**Authors:** Roba El Zibaoui, Yewande E Odeyemi, Mohamad El Labban

**Affiliations:** 1 Medicine, School of Medicine, American University of Beirut, Beirut, LBN; 2 Pulmonary and Critical Care Medicine, Mayo Clinic, Rochester, USA; 3 Internal Medicine, Mayo Clinic, Mankato, USA

**Keywords:** thoracentesis, chest tube, intrapleural fibrinolytic therapy, asymptomatic pleural effusion, parapneumonic effusion, community-acquired pneumonia

## Abstract

A pleural effusion is an accumulation of fluid in the pleural space due to an imbalance between formation and removal. They're commonly caused by heart failure or infections. We report a case of a 56-year-old male with community-acquired pneumonia and a trace pleural effusion on presentation. Despite clinical improvement with antibiotic therapy, the effusion significantly increased on day two. This case report is unique because the patient had an enlarging effusion, but remained asymptomatic and denied worsening shortness of breath, chest pain, or cough. The patient was treated successfully with chest tube placement and intrapleural fibrinolytic therapy. This report emphasizes the importance of repeat imaging for asymptomatic parapneumonic effusions (PPE) that can complicate community-acquired pneumonia. We aim to raise awareness of the atypical presentation and management of parapneumonic effusions through a case report.

## Introduction

The pleural sac is filled with a thin layer of fluid that enters the pleural space from systemic capillaries in the parietal pleurae and exits via parietal pleural stomas and lymphatics into the right atrium. Any imbalance between the formation and removal of this fluid can lead to the formation of a pleural effusion (PE) [1]. Pleural effusions are almost always a manifestation of an underlying pathology. The signs and symptoms of PE can range from asymptomatic to severe respiratory decompensation, depending on the size, rate of accumulation, and etiology of the effusion [2]. The clinical presentation, diagnostic imaging, and pleural fluid analysis provide important clues to the underlying diagnosis. Pleural effusions are commonly classified as either transudative or exudative effusions. Differentiating between a transudative and exudative effusion is pivotal in identifying the underlying process behind how pleural fluid accumulates. A transudate effusion is almost always associated with an imbalance of fluid or protein throughout the body. It can be produced from fluid overload due to increasing hydrostatic pressure forcing the fluid out of the capillaries and into the extravascular space or decreased oncotic pressure in the capillaries, which can lead to fluid accumulation, typically found in low albumin states such as in nephrotic syndrome or liver failure. An exudative effusion is usually caused by a disease localized to the pleura, such as infection or malignancy that alters capillary permeability and leads to fluid leakage [2]. A pleural effusion can be termed massive when it fills almost all the hemithorax. Massive effusions often suggest an underlying malignancy [3,4]. Treating the underlying cause is the typical management strategy that follows [1]. Pleural effusions of varying severity occur in 40-60% of bacterial pneumonia patients. This report presents a unique case of a rapidly accumulating asymptomatic pleural effusion.

## Case presentation

**Figure 1:**
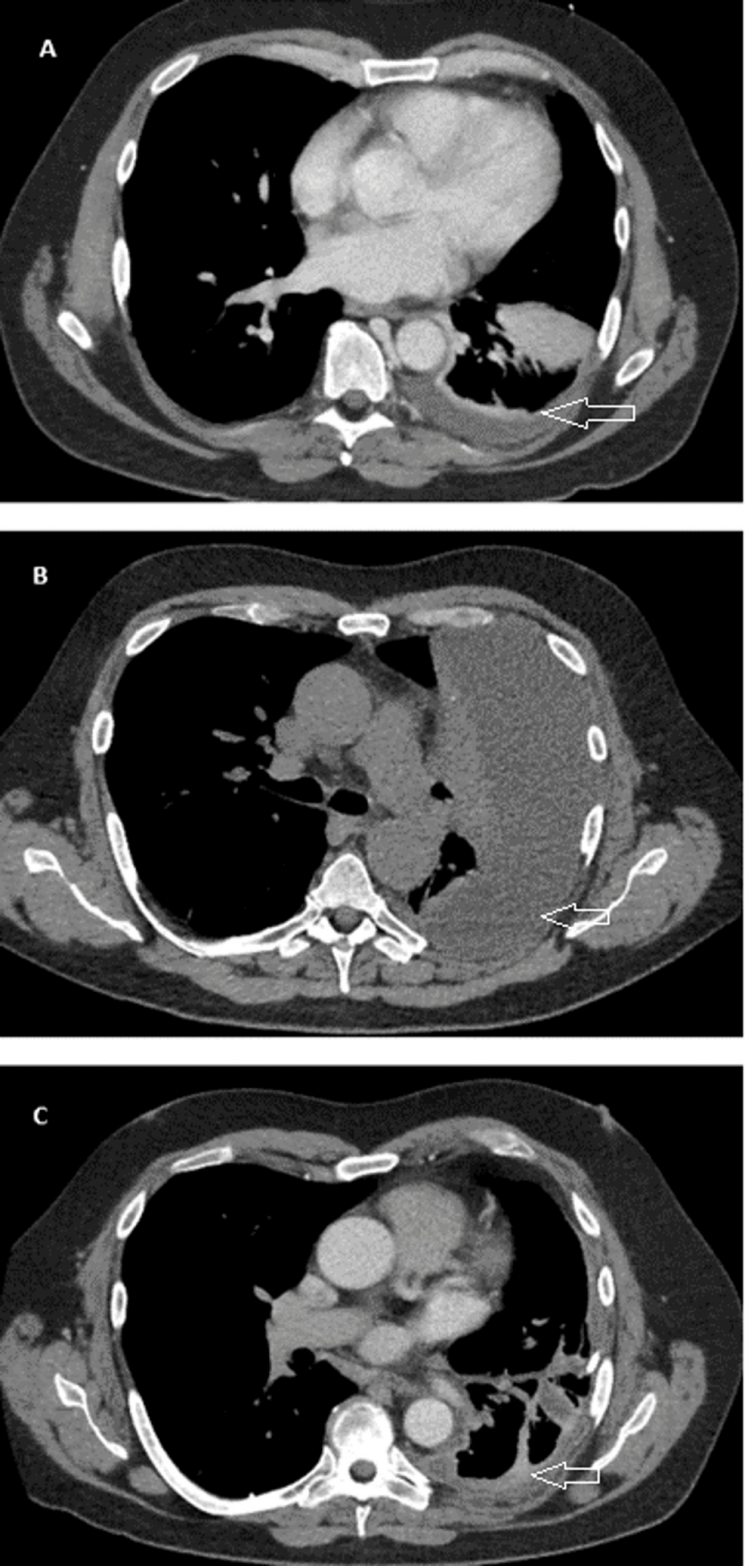
Serial CT imaging A: Day 0: left lower lobe consolidation with trace effusion. B: Day 5: development of a large effusion. C: Day 16: decreased effusion with thick septations. Arrows point to the patient's pleural effusion.

A 56-year-old male patient with a past medical history of hives and obesity class II presented to the Emergency Department with leg swelling and shortness of breath. Two days before the presentation, the patient woke up with left leg swelling, erythema, and pain. The patient denied any recent leg trauma or prolonged immobilization, including recent travel. He also had a left upper quadrant abdominal that was worse with deep inspiration, sharp, and radiating to the left flank. Other associated symptoms included shortness of breath and left-sided chest pain that worsened with inspiration. He denied associated cough, orthopnea, recent upper respiratory illness, and other systemic symptoms. Workup was notable for neutrophilic leukocytosis (WBC 12.9 x10(9)/L), elevated procalcitonin (8.81 ng/ml), mildly elevated liver function tests (aspartate aminotransferase (AST) 64 U/L and alanine aminotransferase (ALT) 77 U/L), and sinus tachycardia on an electrocardiogram. A venous Doppler left leg as well as a CT angiogram of the chest were negative for deep vein thrombosis and pulmonary embolism, respectively. The CT chest (Figure 1A), however, did show evidence of left lower lobe consolidation, and hence, the patient was discharged home on cefdinir, treating community-acquired pneumonia.

**Figure 2:**
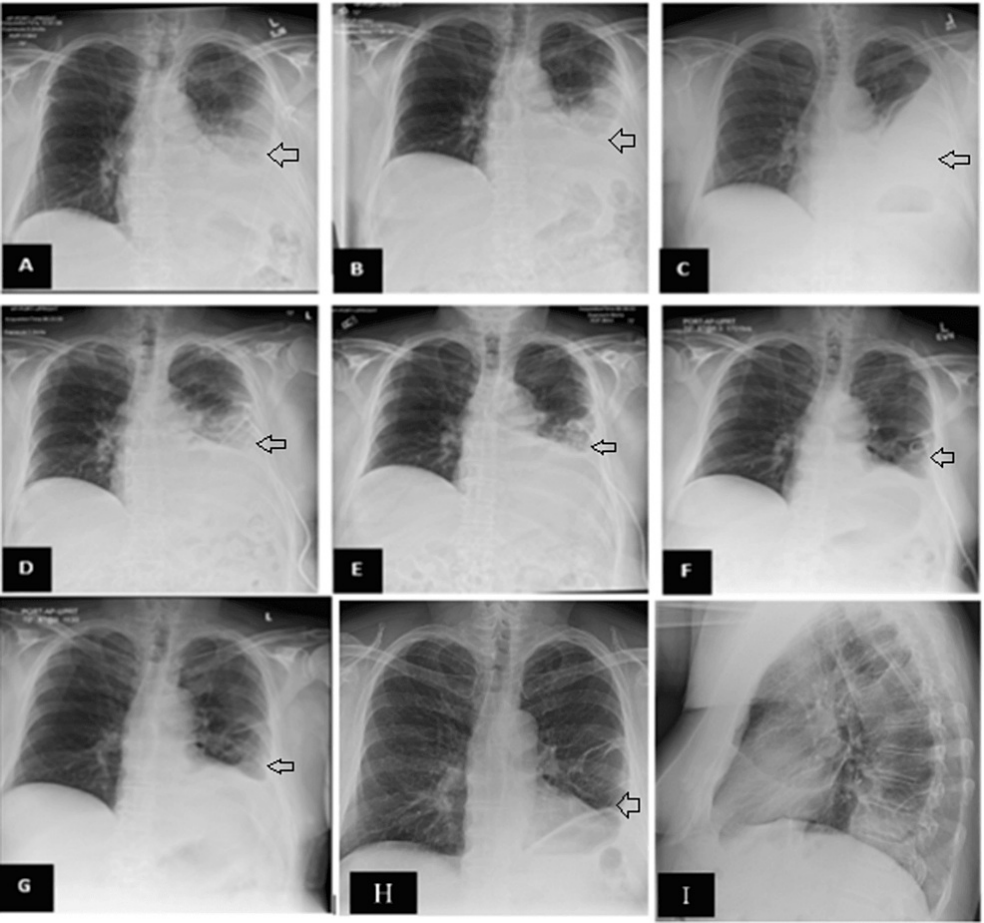
Serial Chest X-rays (A) Day two. (B) Day two post thoracentesis. (C) Day four. (D) Day six following placement of chest tube. (E) Day nine following completion of fibrinolytic therapy. (F) Day 15 following placement of a larger caliber chest tube. (G) Day 17 after removal of a chest tube. (H) Day 26 post-discharge showing a small residual effusion. (I) Day 26 post-discharge lateral chest x-ray. Arrows point to the patient's pleural effusion.

One day later, the patient returned to the Emergency Department for increased pain and swelling in the left leg, a fever of 38.3 °C, and worsening chest pain. A review of systems was otherwise unremarkable. A physical exam revealed a temperature of 36.4 °C, pulse rate of 105 beats/minute, respiratory rate of 24 breaths/minute, blood pressure of 150/107 mmHg, and an oxygen saturation of 96% on room air. The patient was not in respiratory distress; he had symmetrical breathing sounds bilaterally without added sounds. The abdomen was soft and non-tender. The left lower extremity had pitting edema up to the knee with overlying erythematous and tender skin. A CT abdomen pelvis with contrast showed left lower lung lobe consolidation with trace left pleural effusion but did not reveal any intra-abdominal pathology. The patient was admitted for further management of pneumonia and cellulitis, and antibiotics transitioned to ceftriaxone and doxycycline. On day two, the patient’s symptoms of chest/left upper abdominal pain started improving, and a transthoracic echocardiogram showed regional wall motion abnormalities with a normal ejection fraction (EF). On day three, cardiac magnetic resonance imaging showed a left ventricular EF of 46%, a right ventricular EF of 45%, and no abnormal myocardial delayed enhancement to suggest inflammation, infiltration, or ischemia. The remarkable finding, however, was the detection of an enlarging left pleural effusion. A subsequent chest x-ray (CXR) (Figure 2) showed an increasing moderate size left pleural effusion with left midlung and lower lobe consolidating infiltrates. This was an unexpected finding, given the significant improvement in pleuritic chest pain and shortness of breath. Bedside ultrasound revealed a loculated left pleural effusion with multiple septations.

The antibiotic coverage was broadened with piperacillin/tazobactam for anaerobic coverage for concerns of empyema, and a thoracentesis drained 260 ml of pleural fluid with analysis significant for an exudative effusion (Table 1). 

**Table 1:** The patient's pleural fluid analysis LDH: lactate dehydrogenase, PCR: polymerase chain reaction

Test	Normal Range	Thoracentesis Day Three	Thoracentesis Day Five
Amount		260 ml	150 ml
Gross Appearance		Straw-colored	Serous yellow
Total Nucleated Cells	<500 /mcL	5901	450
Neutrophils	<25%	94	65
Lymphocytes		1	15
LDH (U/L)		1254	607
LDH (plasma)	122-222 U/L	169	-
Total protein (g/dl)		3.8	4
Total protein (Plasma)	6.3-7.9 g/dl	5.6	5.4
Lipase (U/L)		-	12
Triglycerides (mg/dl)	<110	-	52
Albumin (g/dl)		-	2.2
Creatinine (mg/dl)		-	0.8
Glucose (mg/dl)		71	84
pH		-	7.42
Gram Stain		No organisms seen	No organisms seen
Bacterial Culture		No growth	No growth
Fungal Culture		No growth	No growth
Broad Range Bacteria PCR + Sequencing		-	No bacterial DNA was detected.
Acid Fast Smear for Mycobacterium		-	Negative

The left pleural fluid was loculated, septated, and could not be completely aspirated on ultrasound. The pleural fluid analysis is further described in Table 1. Pleural fluid bacterial and fungal cultures and broad range bacterial polymerase chain reaction (PCR) were negative for any growth. On day four, the patient continued to improve clinically and, at that point, did not complain of any chest pain or shortness of breath. The leukocytosis (9.4 x 10(9)/L) and procalcitonin improved (3.44 ng/ml). A repeat CXR (Figure 2C) showed stable, moderately large, partially loculated left pleural effusion. On day five, Pulmonary Medicine ordered a CT chest (Figure 1B) that showed an unchanged large loculated left pleural effusion with associated compressive atelectasis. Consequently, on day five, a left chest tube was placed under ultrasound guidance, draining 150 ml of yellow clear fluid (Table 1). The pleural fluid analysis on day five showed a normal pH and a decrease in total nucleated cell count compared to the initial analysis, which does not suggest an empyema diagnosis. Intrapleural fibrinolytic-DNase therapy (alteplase 10 mg/dornase 5 mg) on day six was started. Due to intolerability, specifically chest pain, an adjusted regimen of alteplase 5 mg/dornase 2.5 mg was given twice daily for a total of six doses. Over the next 24 hours, the tube drained over 3 liters of pleural fluid. The chest tube remained on suction with daily chest X-rays (Figure 2E) showing a small persistent left pleural effusion; consequently, a chest tube with a larger caliber was placed on day 14. Two days later, the CT chest showed a marked interval size decrease in the pleural effusion (Figure 1C). At that point, the patient continued to be asymptomatic with decreasing drainage; therefore, the chest tube was discontinued on day 16. The patient completed 14 days of antibiotics. Nine days after discharge from the hospital, the patient was seen in the outpatient pulmonary clinic. He was doing well and denied shortness of breath, chest pain, cough, fever, chills, or lower extremity edema. Examination revealed normal breath sounds bilaterally with no crackles. Repeat chest imaging (Figure 2H, 2I) done 26 days after discharge revealed a small residual pleural effusion with pleural thickening.

## Discussion

Pleural effusions may initially be present with or without associated signs and symptoms. Patients may present with non-specific dyspnea, cough, fever, and pain. A physical exam may reveal a pleural friction rub, absent tactile fremitus, dullness to percussion, and decreased breathing sounds [1]. Large pleural effusions are seldom asymptomatic [5]. Symptoms such as dyspnea and cough accompany most pleural effusions and commonly worsen as the effusion increases. This is often due to several factors, such as decreased chest wall compliance, reduced ipsilateral lung volume, contralateral shifting of the mediastinum, and the stimulation of pleural or airway cough receptors [6]. Chest pain can be either sharp or dull and can localize to an area of pleura or present as referred pain in the abdomen and the ipsilateral shoulder. Nonetheless, pain at distal sites occurs due to the intercostal innervation above and below the diaphragm stimulated by larger effusions [6]. Common causes of pleural effusions are mentioned below (Table 2).

**Table 2:** Common Causes of Pleural Effusions Characterization by Light criteria and clinical features modified from [2]

Transudative Pleural Effusions	Exudative Pleural Effusions
Frequent	
Congestive Heart Failure Liver cirrhosis Hypoalbuminemia Peritoneal dialysis	Malignancy Parapneumonic effusions
Less Common	
Hypothyroidism Nephrotic syndrome Mitral stenosis Pulmonary embolism	Pulmonary infarction Autoimmune diseases Benign asbestos effusion Pancreatitis Post-myocardial infarction syndrome
Rare	
Constrictive pericarditis Superior vena cava obstruction Ovarian hyperstimulation	Drugs Fungal infections

This differential can be made through a thoracentesis and pleural fluid analysis (Table 3) [7].

**Table 3:** Characteristics of Pleural Fluid LDH: lactate dehydrogenase modified from [2]

Parameter	Transudative Pleural Effusions	Exudative Pleural Effusions
Appearance	Colorless to yellow	Cloudy, Purulent, Opaque
Cells	<1,000/mm^3^	>1000/mm^3^
Specific Gravity	<1.012	>1.020
PH	>7.2	<7.2
Glucose	>40 mg/dl	<40 mg/dl
Protein	< 3g/00 ml	> 3g/00 ml
LDH	Low< 200 IU/L	High >200 IU/L
Light’s Criteria		
Effusion Protein/serum protein ratio	< 0.5	> 0.5
Absolute LDH level in pleural fluid	< 200 IU/L	> 200 IU/L
Pleural fluid LDH/serum LDH ratio	< 0.6 or <2/3	> 0.6 or >2/3

In our report, the patient had pain in the left upper quadrant extending back to the left flank. However, this pain was at presentation when the initial CT imaging showed only trace effusions. Despite a growing pleural effusion, the pain continued to improve on medical management with antibiotics. Hence, the pleuritic pain was likely secondary to the pneumonia rather than the pleural effusion. Although asymptomatic pleural effusions are uncommon, they are often described as small and transudative [8]. This is because transudative effusion, such as in postpartum or left ventricular failure, often lacks the primary inflammatory process accompanying exudative or parapneumonic effusions (PPE) to stimulate the receptors. Moreover, small effusions, even bilateral ones, may not be sufficient to compress airways significantly to lead to symptoms [5,6].

Pneumonia is one of the most common causes of hospitalizations in the US. Around 1.4 million visits per year to the ED have a diagnosis of pneumonia, with an annual mortality rate of about 40,000 patients [9]. At least 40% of all patients with pneumonia develop pleural effusions, although only a minority will require intervention for a complicated effusion or empyema [10]. Most pleural effusions, whether transudative or exudative, often progress slowly over a few days to weeks, depending on the underlying etiology. This has been attributed to the relatively low rate of pleural fluid production compared to the 20-fold excess drainage capacity of the pleural space [11]. Despite a growing pleural effusion, our patient was improving, which contrasts with other cases with rapidly growing PPE reporting clinical deterioration. Explosive pleuritis is a pleural effusion that significantly increases in size in less than 24 hours and is associated with rapid clinical deterioration. All reported cases of explosive pleuritis have been symptomatic PPE [3,12] [13-16]. Positive pleural fluid cultures for streptococcus were found in 30 to 40% of patients [12].

As the etiology of PE is quite broad and heterogeneous, the treatment of choice is often related to the underlying cause. Transudative effusions typically resolve spontaneously without treatment once the underlying conditions have been resolved. The management of PPE depends on the clinical and radiological presentation. An uncomplicated, free-flowing, sterile PPE can often be resolved by managing the underlying pneumonia. On the other hand, empyema (frank pus in the pleural space) and complicated PPE often require drainage. Other indications for drainage include large free-flowing effusions (i.e., ≥ hemithorax), effusions associated with thickened parietal pleura, and sepsis from a suspected pleural source. Chest tubes remain in place until drainage is below 50 ml/day and any cavities have closed [10,17]. Non-surgical approaches are increasingly used for refractory parapneumonic effusion management. A study of over 4000 patients with empyema reported a 55% response rate to nonsurgical therapies, including antibiotics, thoracostomy tubes, and fibrinolytics [18]. In this report, despite broadening antibiotics and initial thoracentesis, fibrinolytic therapy was started as the loculated effusion didn’t improve. Previous reports suggest that significant organization and pleural thickening may correlate with poor response to fibrinolytic therapy [19]. A study published in the New England Journal of Medicine showed that a combination of intrapleural t-PA-DNase improved outcomes (increased fluid drainage, decreased frequency of surgical referral, and length of hospital stay) compared to placebo and either therapy alone [20]. Intrapleural bleeding is the main limitation of lytic therapy; however, the occurrence of such adverse events is not common as reported in the study (Serious adverse events: t-PA-DNase 6% vs. Placebo 2%, p-value 0.22 by Fisher’s exact test) [20]. The bleeding risk increases with repeated t-PA-DNase therapy. The use of fibrinolytic-DNase therapy resulted in a significant improvement in our patient's PPE.

This case report offers several lessons. First, a large asymptomatic exudative pleural effusion is an uncommon diagnosis. Our patient had a rapidly enlarging effusion but remained asymptomatic. Second, not all parapneumonic effusions need to be drained. In our patient, the effusion was loculated and enlarging despite antibiotic therapy. Third, t-PA-DNase therapy has decreased the need for surgical interventions in complex parapneumonic effusions. The limitation of this study is the lack of repeat imaging after discharge for objective resolution of the effusion, although the clinical improvement was remarkable.

## Conclusions

Parapneumonic effusions are common complications of community-acquired pneumonia. Most PPE are small and resolve with antibiotic therapy. Enlarging PPE are usually symptomatic and might require drainage with or without fibrinolytic therapy. Asymptomatic presentation of an enlarging effusion is uncommon. Therefore, it is crucial to consider chest imaging before discharging a patient with pneumonia.
